# Low plasma serotonin linked to higher nigral iron in Parkinson’s disease

**DOI:** 10.1038/s41598-021-03700-2

**Published:** 2021-12-21

**Authors:** Leslie C. Jellen, Mechelle M. Lewis, Guangwei Du, Xi Wang, Martha L. Escobar Galvis, Stanislaw Krzyzanowski, Colt D. Capan, Amanda M. Snyder, James. R. Connor, Lan Kong, Richard B. Mailman, Patrik Brundin, Lena Brundin, Xuemei Huang

**Affiliations:** 1 Department of Neurology, Penn State University-Milton S. Hershey Medical Center, Hershey, PA USA; 2 Public Health Sciences, Penn State University-Milton S. Hershey Medical Center, Hershey, PA USA; 3 Parkinson’s Disease Center, Department of Neurodegenerative Science, Van Andel Institute, 333 Bostwick Ave NE, Grand Rapids, MI 49503 USA; 4 Department of Pharmacology, Penn State University-Milton S. Hershey Medical Center, Hershey, PA USA; 5 Division of Psychiatry and Behavioral Medicine, Michigan State University College of Human Medicine, Grand Rapids, MI USA; 6 Departments of Neurosurgery and Radiology, Penn State University-Milton S. Hershey Medical Center, Hershey, PA USA; 7 Department of Kinesiology, Penn State University-Milton S. Hershey Medical Center, Hershey, PA USA; 8 Translational Brain Research Center, Penn State University-Hershey Medical Center, 500 University Dr., Mail Code H037, Hershey, PA 17033 USA

**Keywords:** Diseases, Medical research, Neurology

## Abstract

A growing body of evidence suggests nigral iron accumulation plays an important role in the pathophysiology of Parkinson’s disease (PD), contributing to dopaminergic neuron loss in the substantia nigra pars compacta (SNc). Converging evidence suggests this accumulation might be related to, or increased by, serotonergic dysfunction, a common, often early feature of the disease. We investigated whether lower plasma serotonin in PD is associated with higher nigral iron. We obtained plasma samples from 97 PD patients and 89 controls and MRI scans from a sub-cohort (62 PD, 70 controls). We measured serotonin concentrations using ultra-high performance liquid chromatography and regional iron content using MRI-based quantitative susceptibility mapping. PD patients had lower plasma serotonin (*p* < 0.0001) and higher nigral iron content (SNc: *p* < 0.001) overall. Exclusively in PD, lower plasma serotonin was correlated with higher nigral iron (SNc: r(58) =  − 0.501, *p* < 0.001). This correlation was significant even in patients newly diagnosed (< 1 year) and stronger in the SNc than any other region examined. This study reveals an early, linear association between low serotonin and higher nigral iron in PD patients, which is absent in controls. This is consistent with a serotonin-iron relationship in the disease process, warranting further studies to determine its cause and directionality.

## Introduction

Parkinson’s disease (PD) is a neurodegenerative disorder characterized clinically by bradykinesia, rigidity, and/or tremor and pathologically by dopaminergic neuron loss in the substantia nigra pars compacta (SNc) and the presence of ⍺-synuclein-containing inclusions in cell bodies or neurites (Lewy pathology)^1,2^. PD also involves varying extents of extra-dopaminergic pathology in regions beyond the basal ganglia, and patients can suffer from many non-motor symptoms, not all of which are directly attributed to loss of dopamine^3^. The etiology and pathogenesis of PD remain poorly understood, but multiple genetic, environmental, and aging-related factors are thought to contribute to its pathophysiology^4^.

Iron accumulation in the brain is common with aging and consistently reported to be increased in PD^5^, particularly in the substantia nigra^6–8^ (and specifically the SNc^9–14^). This accumulation has been suggested to contribute to dopaminergic neuron loss via multiple mechanisms. Although iron is essential to many biochemical processes, iron overload can predispose cells to oxidative stress^15^, ferroptosis^16^, and neuroinflammation^17,18^. In dopaminergic neurons, excess iron can be particularly damaging as iron-dopamine interactions can additionally facilitate the formation of highly neurotoxic dopamine intermediates^18^. Iron also interacts directly with ⍺-synuclein, and a complex interplay between these two factors in PD has been proposed to exacerbate ⍺-synuclein aggregation and iron-mediated damage^19,20^. Nigral iron content can be estimated in vivo using MRI-based susceptibility imaging, including state-of-the-art quantitative susceptibility mapping (QSM)^6,21^. Recent studies show that QSM sensitively and reliably reflects increased nigral iron content^6,9,21–23^ and that nigral QSM values are consistently higher in PD patients compared to controls overall^8^. In PD, higher nigral QSM values are associated with increased age and motor severity^9^ with some studies reporting associations with longer disease duration^8^. Large inter-individual differences in nigral iron content among PD patients also exist^24,25^, the causes of which remain insufficiently understood.

Serotonergic neurodegeneration and dysfunction, including low serotonin (5-hydroxytrypamine, or 5-HT) concentrations in plasma^26^, CSF^27^, and several regions of the brain (measured post-mortem)^28^, also can be present to varying extents in PD patients^29,30^, even very early in the disease^31,32^. Low serotonin is thought to contribute to the high rate (~ 35%) of clinically significant depression, an often prodromal symptom, among patients^33,34^. Consistent with this, lower plasma, serum, and CSF concentrations of serotonin and/or its metabolite, 5-hydroxyindoleacetic acid (5-HIAA) in PD have been associated with depression^35,36^ or higher depression scores^26,37^ (but see^38^). Lower CSF serotonin also has been associated with freezing of gait^27^.

Although serotonergic dysfunction and iron dyshomeostasis typically have been studied as independent disease features in PD, several lines of converging evidence suggest they might be closely related, if not directly contributing to each other. First, the serotonin transporter (SERT) has been suggested to regulate ventral midbrain iron concentrations^39^. Specifically, genetic variation leading to SERT dysfunction was associated with higher ventral midbrain iron concentrations in affected recombinant inbred mouse strains, and SERT knockouts showed selective decreases in iron content in the same region^39^. Interestingly, SERT is a major determinant of plasma serotonin concentrations^40^ and its expression/activity are altered by proteins disrupted in PD, including ⍺-synuclein^41^ and proinflammatory TNF-⍺^42^. Secondly, in CSF, low serotonin concentrations in PD patients are correlated with higher concentrations of transferrin, an iron transport protein that regulates iron influx^37^. In addition, low serotonin and higher iron and transferrin in CSF are associated with mental fatigue, a symptom associated with higher depression scores^37^. Third, higher depression scores and freezing of gait, two factors associated with low plasma and/or CSF serotonin, also are associated with higher nigral iron in PD patients^43,44^. The depression-nigral iron association is present even in patients with mild symptom severity, implying an early relationship in the disease^43^.

In the present study, we tested the hypothesis that lower serotonin would be associated with higher nigral iron in PD patients, even in early-stage disease. We measured plasma serotonin concentrations in PD and control subjects and determined their association with QSM values in the SNc, first in both groups, then in PD by disease stage. We then explored their associations with QSM values in other regions and with clinical outcomes.

## Results

### Demographic and clinical characteristics of subjects

We obtained plasma samples and clinical data from *n* = 186 participants in the NINDS PD biomarker program (97 PD patients, 89 controls), whose demographic and clinical characteristics are summarized in Table 1. MRI scans were obtained from a subset of these subjects who consented and were able to complete the scan with good image quality (*n* = 62 PD, 70 controls). In both the full cohort and MRI sub-cohort, PD patients and controls were similar in age, sex, BMI, and years of education and showed expected differences in PD-related metrics. Notably, PD patients had a four-fold higher rate of SSRI/SNRI use, indicating treatment for depression/anxiety, than controls overall, with approximately one-third of patients reporting use of an SSRI or SNRI (32% vs. 8%; *p* < 0.001).

**Table 1:** Demographic and clinical characteristics of the cohort.

Number of Subjects	Entire Cohort (*N* = 186)			MRI Cohort (*N* = 132)		
	Controls	Parkinson’s	Group comparisons (*p*-value)	Controls	Parkinson’s	Group comparisons (*p*-value)
	89	97		70	62	
**Demographics**						
Females (n, %))	47 (53%)	48 (49%)	0.650^a^	38 (54%)	32 (52%)	0.759^a^
Age	66.6 ± 10.3	67.0 ± 8.3	0.790^b^	66.1 ± 10.3	65.8 ± 8.1	0.853^b^
BMI	28.0 ± 4.7	27.2 ± 4.9	0.272^b^	27.5 ± 4.6	26.7 ± 4.8	0.376^b^
Education (years)	15.4 ± 2.6	14.8 ± 2.6	0.129^b^	15.4 ± 2.6	14.7 ± 2.8	0.131^b^
**Parkinson’s and other clinical metrics**						
Disease duration	–	6.5 ± 6.5	–	–	5.3 ± 4.9	–
LEDD	–	686 ± 450	–	–	701 ± 443	–
MDS UPDRS I	3.7 ± 3.8	9.7 ± 7.2	**< 0.001**^b^	3.5 ± 3.5	7.8 ± 6.0	** < 0.001**^b^
MDS UPDRS II	0.5 ± 1.0	10.3 ± 9.7	**< 0.001**^b^	0.4 ± 0.1	7.6 ± 6.9	** < 0.001**^b^
MDS UPDRS III	4.7 ± 5.5	27.2 ± 20.1	** < 0.001**^b^	4.2 ± 4.1	21.7 ± 13.3	** < 0.001**^b^
MDS UPDRS IV	–	2.3 ± 3.2	–	–	2.4 ± 3.1	–
FOG-Q	–	6.1 ± 5.4	–	–	5.2 ± 4.8	–
UPSIT	32.1 ± 6.5	19.2 ± 7.5	** < 0.001**^b^	44.2 ± 5.5	61.4 ± 20.8	** < 0.001**^b^
PDQ-39	44.6 ± 7.7	67.3 ± 4.7	** < 0.001**^b^	32.8 ± 6.2	19.0 ± 7.8	**< 0.001**^b^
MOCA	25.4 ± 2.4	23.8 ± 3.8	** < 0.001**^b^	25.5 ± 2.5	24.5 ± 3.0	**0.047**^b^
ESS	5.8 ± 3.8	7.9 ± 4.7	**0.001**^b^	5.8 ± 3.6	7.1 ± 4.4	0.076^b^
**Depression-related metrics**						
SSRI/SNRI use	7 (8%)	31 (32%)	**< 0.001**^a^	4 (6%)	16 (26%)	**0.001**^a^
HDRS	2.7 ± 3.2	6.1 ± 4.9	**< 0.001**^b^	2.8 ± 3.3	5.1 ± 4.5	**0.002**^b^
HARS	4.0 ± 4.3	8.2 ± 6.2	**< 0.001**^b^	4.2 ± 4.4	6.9 ± 5.8	**0.003**^b^

The data for the entire cohort (89 controls, 97 Parkinson’s) and subset with MRI data (MRI cohort, 70 controls, 62 Parkinson’s). Data reflect mean ± SD unless otherwise indicated. Two-tailed p-values indicate results of chi-square test^a^ or independent-sample t-test^b^.

*BMI* body mass index, *LEDD* levodopa equivalent daily dosage, P*DQ-39* 39 Item Parkinson’s Disease Questionnaire, *MDS UPDRS* Movement Disorders Society Unified Parkinson’s Disease Rating Scale, *I* non-motor experiences of daily living, *II* motor experiences of daily living, *III* motor examination, *IV* motor dysfunction, *FOG-Q* Freezing of Gait Questionnaire, *PDQ-39* 39 Item Parkinson’s Disease Questionnaire, *UPSIT* University of Pennsylvania Smell Identification Test, *MOCA* Montreal Cognitive Assessment, *ESS* Epworth Sleepiness Scale, *HDRS* Hamilton Depression Rating Scale, *HARS* Hamilton Anxiety Rating Scale.

Significant values are in bold.

### Group differences in plasma serotonin concentrations

**Figure 1:**
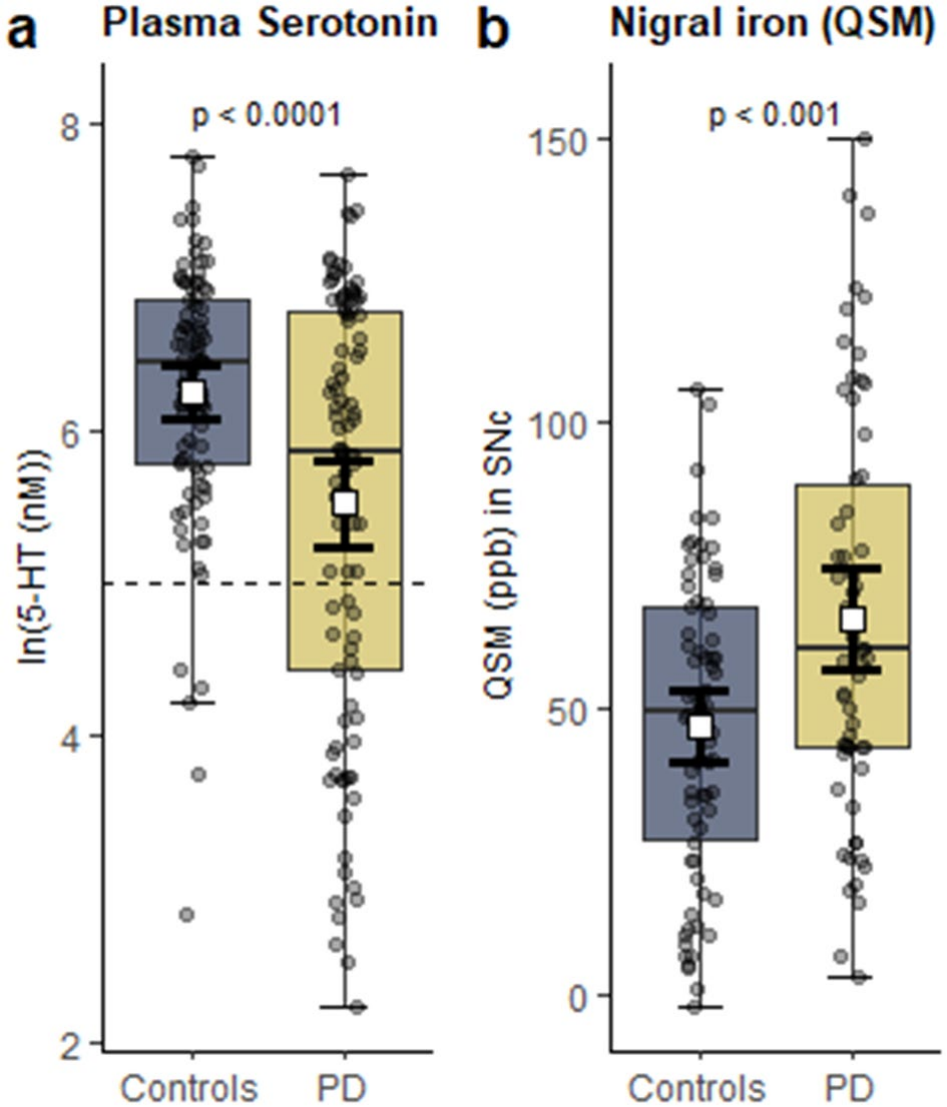
Group differences in (**a**) plasma serotonin (5-HT) concentrations and (**b**) nigral iron content (reflected by QSM values) between PD patients and controls. *P*-values indicate results of independent samples t-tests (two-tailed). White box and error bars indicate mean and 95% CI. Box and whisker plots reflect median and interquartile range. Dashed horizontal line marks lowest tertile of serotonin concentrations in PD group. Figure was produced using the ggplot2 package^80^ in R (version 3.5.3)^81^.

PD patients had lower mean plasma serotonin concentrations than controls [in log scale, 5.53 ± 1.40 vs. 6.25 ± 0.85, *p* < 0.0001; Fig. 1a]; however, the PD distribution appeared distinctly bimodal, and the group difference was driven by approximately one-third of patients with extremely low concentrations (the lowest tertile, *n* = 32; < 150 nM; Supplementary Fig. S1). In the remaining two-thirds of PD patients, plasma serotonin concentrations were distributed relatively widely and within the normal range of controls. Contamination from platelet serotonin can affect measured plasma concentrations, so we compared mean platelet counts between PD patients and controls. There was no significant difference in platelet counts between PD patients and controls (222 ± 50 vs. 230 ± 68; units = 109 platelets/L; *p* = 0.405).

### Group differences in regional iron content in the brain (QSM and R2*) and serum iron status

PD patients had higher mean QSM values than controls in both the SNc [controls: 47.1 ± 26.4 ppb vs. PD: 65.7 ± 35.2 ppb; *p* < 0.001; Fig. 1b] and substantia nigra pars reticulata (SNr) [controls: 132.3 ± 53.4 ppb vs. PD: 161.5 ± 59.6 ppb; *p* = 0.004; data not shown]. QSM values were normally distributed, with a subset of PD patients showing values exceeding the range of controls (Supplementary Fig. S1). PD patients also had higher R2* values than controls in both nigral sub-regions [SNc, controls: 26.0 ± 3.7 ppb vs. PD: 27.6 ± 4.0 ppb; *p* = 0.018 and SNr, controls: 37.0 ± 6.8 ppb vs. PD: 40.6 ± 9.2; *p* = 0.016; data not shown]. In all other regions of interest, there were no statistically significant differences between PD patients and controls in either QSM or R2* values (*p*-values > 0.05; Supplementary Fig. S2). There also were no significant differences in serum iron markers between the groups (Supplementary Table 1).

### Association between plasma serotonin and QSM in the SNc

**Figure 2:**
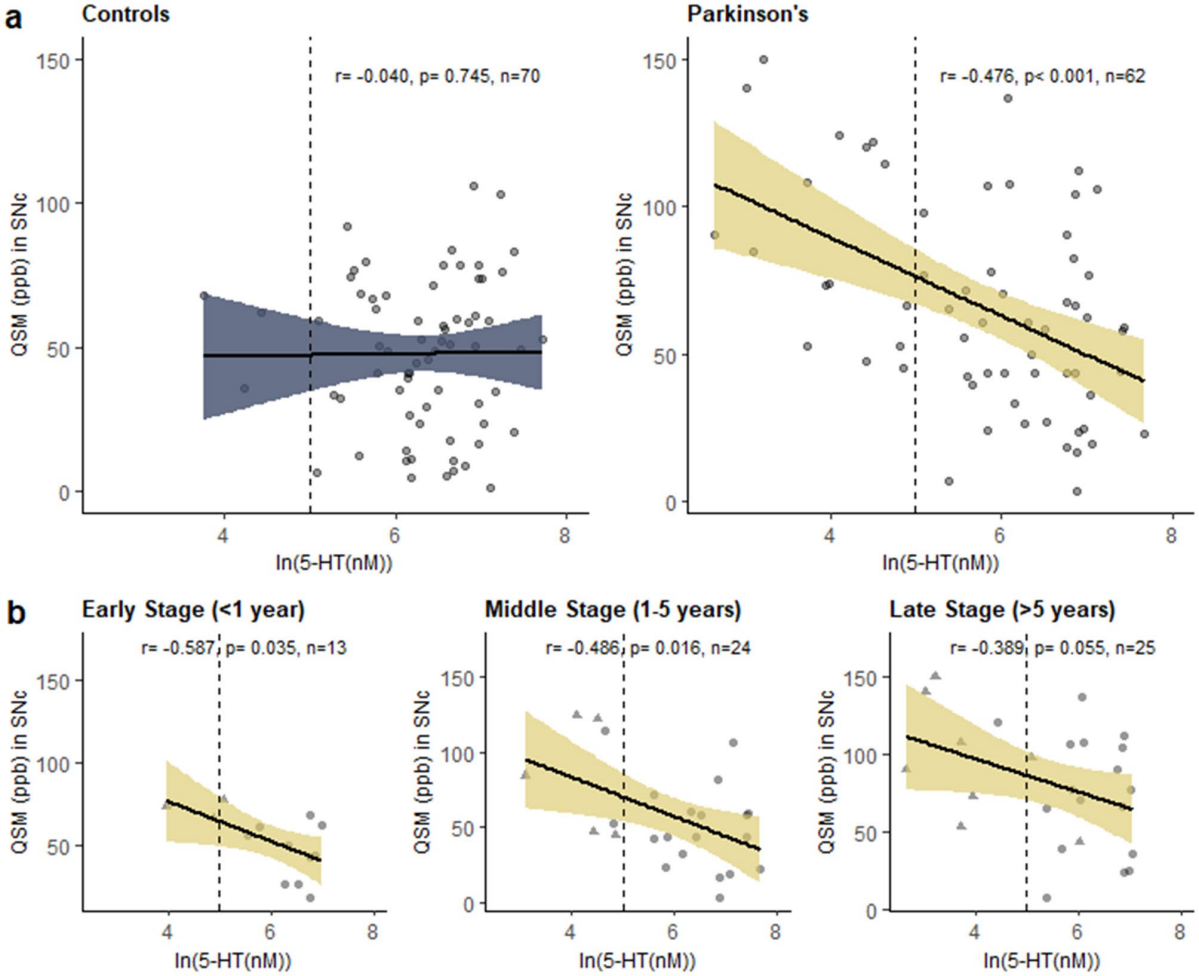
The association between plasma serotonin (5-HT) concentrations and QSM values, reflecting iron content, in the substantia nigra pars compacta (SNc) (**a**) in PD patients and controls, and (**b**) within PD patients by disease stage. Unadjusted Pearson’s r and *p*-values are indicated. Dashed line marks lowest tertile of serotonin concentrations in PD patients. Figure was produced using the ggplot2 package^80^ in R (version 3.5.3)^81^.

Plasma serotonin concentrations were negatively correlated with QSM values in the SNc in PD patients [r(60) =  − 0.476, *p* < 0.001], but not controls [r(68) =  − 0.040, *p* = 0.745; Fig. 2a]. This correlation was strengthened after controlling for age and sex [r(58) = -0.501, *p* < 0.001] and remained significant after additionally controlling for disease duration [r(57) =  − 0.415, *p* < 0.001] and disease duration plus measures of motor severity: LEDD [r(57) =  − 0.362, *p* = 0.005], UPDRS II [r(56) =  − 0.357, *p* = 0.003], UPDRS III [r(56) =  − 0.435, *p* = 0.001]. The association also remained significant after controlling for age, sex, and platelet count [r(56) =  − 0.512, *p* < 0.001]. To assess the correlation across the disease span, we sub-grouped patients by disease stage. The unadjusted correlation between plasma serotonin and QSM in the SNc was statistically significant in early [< 1 year; r(11) =  − 0.587, *p* = 0.035] and middle [1–5 years; r(22) =  − 0.486, *p* = 0.016] stage patients but was only a trend in later stage patients [> 5 years; r(23) =  − 0.389, *p* = 0.055, *n* = 25; Fig. 2b].

### Association between plasma serotonin and R2* in the SNc

R2* results were similar to QSM. There was a negative correlation between plasma serotonin concentrations and R2* values in the SNc in PD patients [r(62) = -0.306, *p* = 0.016] but not controls [r(70) =  − 0.114, *p* = 0.348; data not shown]. To determine the extent to which iron or other PD pathology contributed to this correlation^23^, we determined the correlation between plasma serotonin and R2* after controlling for QSM (the iron component), and vice versa. The correlation between plasma serotonin and SNc R2* in PD patients became negligible after controlling for QSM [r(59) = 0.108, *p* = 0.407], whereas the correlation between plasma serotonin and SNc QSM values remained significant after controlling for R2* [r(59) =  − 0.395, *p* = 0.002].

### Association between plasma serotonin and QSM in other brain regions and with serum iron markers

**Figure 3:**
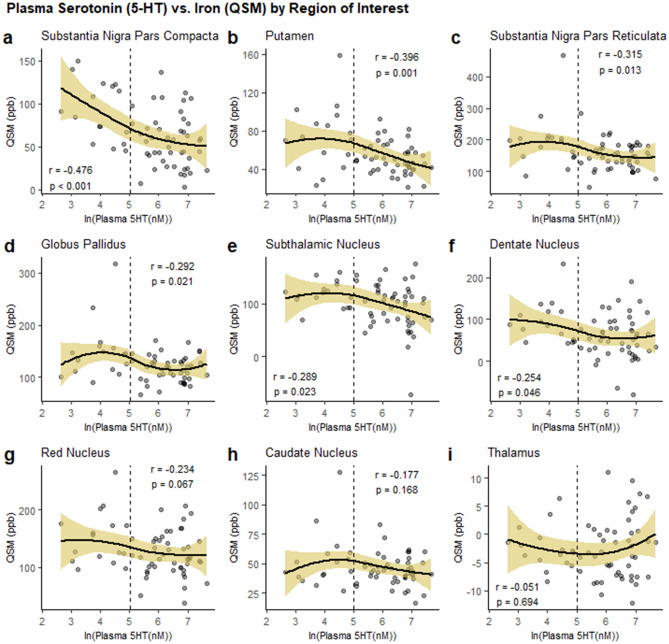
Within PD patients, plasma serotonin’s association with QSM values in the SNc compared to other regions of interest. Loess curves are fitted to the data. Unadjusted Pearson’s correlation coefficients and *p*-values are indicated, uncorrected for multiple comparisons (*N* = 62). Scales on y-axis differ by region based on regional distributions. The dashed line marks the lowest tertile of patients in terms of serotonin concentrations. Figure was produced using the ggplot2 package^80^ in R (version 3.5.3)^81^.

To gauge the regional specificity of the correlation between plasma serotonin and QSM values in the SNc, we explored this association in several other regions of interest. In PD patients, the correlation was strongest in the SNc; however, lower serotonin also was associated with higher QSM values in the putamen [r(62) =  − 0.396, *p* = 0.001], SNr [r(62) =  − 0.315, *p* = 0.013], globus pallidus [r(62) =  − 0.292, *p* = 0.021], subthalamic nucleus [r(62) =  − 0.289, *p* = 0.023], and dentate nucleus [r(62) =  − 0.254, 0.046; Supplementary Table 2, Fig. 3]. The correlation was non-significant in other regions (red nucleus, caudate, thalamus (*p* ≥ 0.067); Fig. 3, Supplementary Table 2). Loess curves for each region (Fig. 3) demonstrated that plasma serotonin and QSM were correlated most robustly in the SNc and that, distinctly in this region, the correlation was strongest among the patients with the lowest serotonin concentrations. In other regions, the correlations seemed to be influenced by a few outliers (Fig. 3). In controls, no correlations were observed between plasma serotonin and QSM in any region (Supp. Table 2). There also were no significant correlations between plasma serotonin and serum iron markers in either PD subjects (Supp. Table 1) or controls (*p*-values > 0.05; data not shown).

### Associations between plasma serotonin and other clinical outcomes

**Figure 4:**
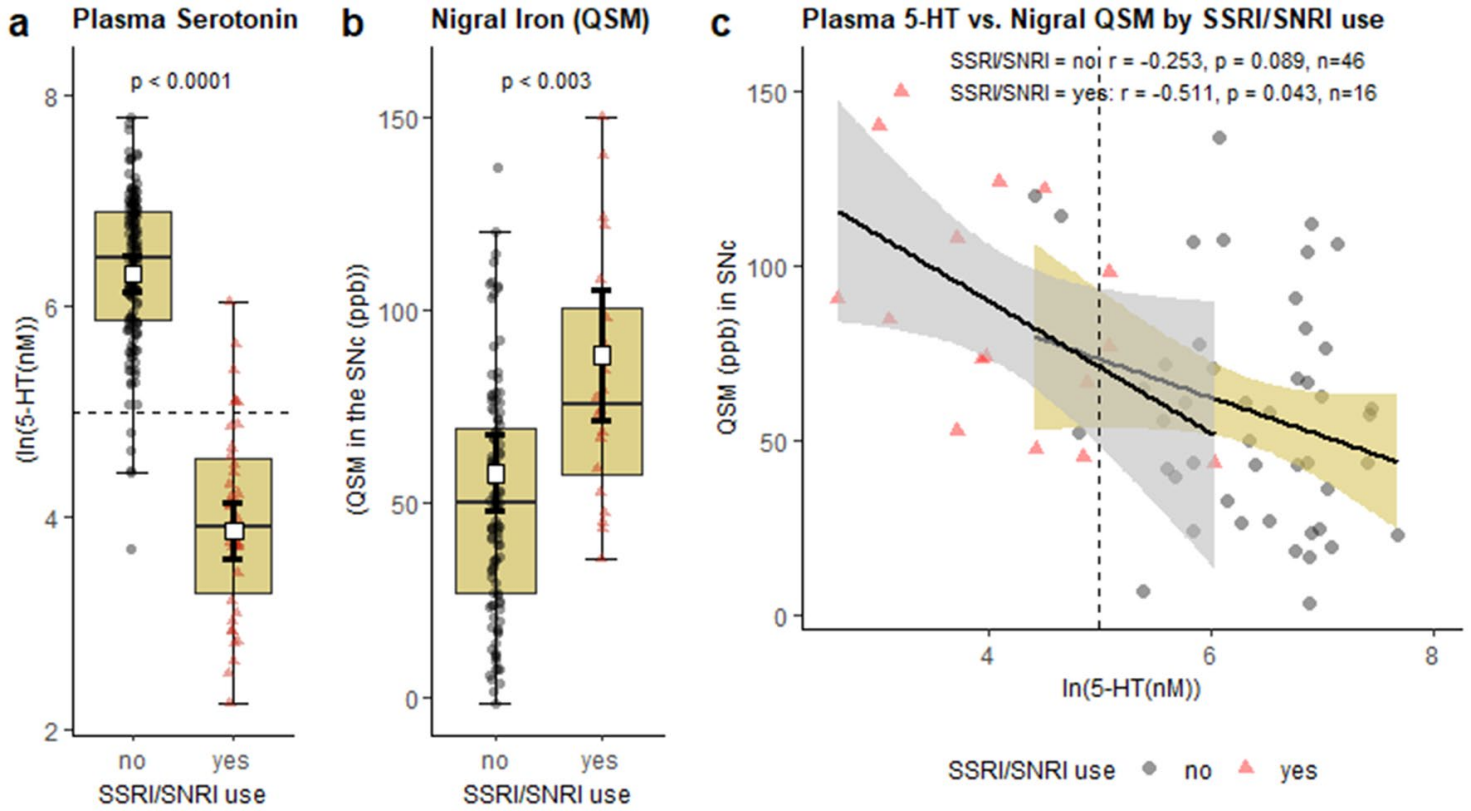
Within PD patients, plasma serotonin and nigral iron (as reflected by QSM values) by SSRI/SNRI use and their association with each other within SSRI/SNRI subgroups (**a**), (**b**). *P*-values indicate results of ANCOVA with age and sex as covariates (*p*-values > 0.05 not shown). Box and error bars indicate age and sex-adjusted mean and 95% CI. (**c**) Unadjusted Pearson’s correlations. Dashed line marks lowest tertile of serotonin concentrations in PD group. Figure was produced using the ggplot2 package^80^ in R (version 3.5.3)^81^.

In PD patients, plasma serotonin concentrations were not associated significantly with age (r = 0.092, *p* = 0.371), sex (t =  − 0.610, *p* = 0.544), or disease duration (r = -0.142, p = 0.165). Lower plasma serotonin concentrations, however, were weakly associated with higher LEDD (r =  − 0.291, *p* = 0.004) and worse scores on UPDRS I (r =  − 0.251, *p* = 0.014), FOG-Q (r =  − 0.278, *p* = 0.006), and PDQ-39 (r =  − 0.341, *p* = 0.001). There were no significant associations between plasma serotonin and measures of motor severity or complications (UPDRS II, III, or IV), hyposmia (UPSIT), cognition (MOCA), or sleepiness (ESS; *p*-values > 0.05). Of the depression-related outcomes, low plasma serotonin was strongly associated with SSRI/SNRI use, in both PD patients [age- and sex-adjusted mean: 3.88 (3.62, 4.14) in users vs. 6.30 (6.12, 6.48) in non-users; *p* < 0.0001; Fig. 4a] and controls [4.35 (3.80, 4.90) in users vs. 6.41 (6.25, 6.57), *p* < 0.0001 in non-users; data not shown)]. There was no relationship between plasma serotonin and depression or anxiety scores (HDRS or HARS) in either group, even after controlling for SSRI/SNRI use (*p*-values > 0.05).

### Associations between nigral iron and other clinical outcomes

Similar to plasma serotonin, QSM values in the SNc were not associated with age or sex in PD patients. In contrast, higher SNc QSM values were associated with longer disease duration (r = 0.465, *p* < 0.001). Clinically, higher QSM values in the SNc were associated with the same outcomes as low plasma serotonin, including higher LEDD (r = 0.450, *p* < 0.001) and worse scores on UPDRS-I (r = 0.304, *p* = 0.016), FOG-Q (r = 0.353, *p* = 0.005), and PDQ-39 (r =  − 0.310, *p* = 0.014). Higher SNc QSM values also were associated with SSRI/SNRI use (*p* = 0.003; Fig. 4b). All clinical associations with plasma serotonin remained significant after controlling for age, sex, and disease duration (Model 1a; Supplementary Table S3) but were weaker in the MRI sub-cohort (Model 1b; Supplementary Table S3) and disappeared or were further weakened by controlling for QSM in the SNc (Model 2; Supplementary Table S3).

### Low plasma serotonin, SSRI/SNRI use, and the influence of SSRI/SNRI use on the serotonin-iron association

Because SSRI/SNRI use was more frequent in PD patients and strongly associated with both lower plasma serotonin and higher QSM values in the SNc, we investigated its potential influence on the serotonin-iron association. Partial correlation analysis showed that the association between plasma serotonin and QSM values in the SNc in PD patients remained significant after controlling for age, sex, disease duration, and SSRI/SNRI use [r(56) =  − 0.356, *p* = 0.006]. In SSRI/SNRI users as a sub-group, plasma serotonin was significantly correlated with QSM values in the SNc [r =  − 0.511, *p* = 0.043, *n* = 16], whereas in SSRI/SNRI non-users the correlation was weaker and there was only a trend toward significance (r =  − 0.253, *p* = 0.089, *n* = 46; Fig. 4c).

## Discussion

The present study investigated the potential relationship between low serotonin and increased nigral iron accumulation in PD. We first demonstrated, consistent with previous studies^8,26^, that PD patients had lower plasma serotonin concentrations and higher nigral QSM values (reflecting higher nigral iron content) than controls overall. Both features, however, showed wide inter-individual variation. Exclusively in PD patients, we observed a negative correlation between plasma serotonin concentrations and iron content in the SNc. Importantly, this correlation existed regardless of correction for age, sex, disease duration, and motor symptom severity (LEDD, UPDRS II and III), suggesting low serotonin and higher nigral iron are not simply clustering together in males or females or worsening in parallel as patients get older or the disease progresses. In fact, the correlation appeared strongest in patients during the first year of diagnosis but waned in later stage disease, when an increasing number of influences on these features presumably come into play. We also observed correlations between plasma serotonin and QSM in several other brain regions; however, none of these correlations were as robust as the one involving the SNc. Clinically, worse outcomes on several metrics were weakly associated with both low serotonin and higher nigral iron. These included freezing of gait, a symptom independently tied to each of these features in past studies^27,44,45^. Notably, SSRI/SNRI use, indicating treatment for depression or anxiety, was strongly associated with both lower plasma serotonin and higher nigral iron, but did not explain the correlation between these two features. Taken together, our findings reveal an early, robust association between low serotonin and higher iron in the SNc in PD and weaker associations between low serotonin and higher iron content in several other regions. We propose this lends further support for a serotonin-iron relationship in the disease process.

To our knowledge, this is the first evidence linking low serotonin to higher nigral or other regional iron content in the brain in PD. Our findings are consistent mechanistically with prior reports of both an inverse serotonin-transferrin correlation in CSF in PD patients^37^ and a correlation between higher depression and anxiety scores and higher nigral iron content in PD, even in patients with overall mild Parkinson’s symptom severity^43^.

Neither the current nor previous cross-sectional studies allow inference of causality; however, collectively they suggest three intriguing possibilities: (1) that low serotonin contributes to nigral iron accumulation, either early in PD or as a risk factor to developing the disease, (2) that early nigral iron accumulation in the disease contributes to low serotonin (and depression), and/or (3) that another factor (or factors) mediates the relationship between the two. Exploring these possibilities could lend insight into serotonin and iron regulation in the disease and possible novel therapeutic targets.

An important observation in the current study was the absence of any serotonin-iron association in healthy controls, suggesting a PD-specific or disease-specific phenomenon. Interestingly, however, in the general population, major depression is associated with both lower plasma serotonin in older adults^46^ and higher iron content in the putamen (reflected by QSM; the SN was not included as a region of interest)^47^. In the current study, the putamen of PD patients had the second strongest inverse correlation between plasma serotonin and iron content. It is possible that our observation of the serotonin-iron association only in PD patients simply reflects increased power to detect this association due to the relatively high rate of depression (and low plasma serotonin) in the PD population^33,34^. If so, the association could indicate a more general relationship between low serotonin/major depression and iron accumulation in the SNc, putamen, and other regions of the brain. Notably, depression in PD patients often emerges in the few years prior to PD diagnosis and is a risk factor (and possible causal risk factor) for the disease in older adults^48^. That increased iron accumulation in the brain in individuals with low serotonin and depression could mediate this risk is an intriguing possibility.

Another interesting observation was that the serotonin-QSM association was most robust in the SNc, the major site of dopaminergic neuron loss and also the region where increased iron is reported most widely and consistently in PD^8^. Although iron accumulation in PD has been reported in other regions, including the putamen^49^, this occurs more variably. A recent systematic review reports that extra-nigral iron accumulation in PD is less consistently observed across QSM studies, with only a minority of studies reporting increases in extra-nigral regions and some studies reporting decreases^8^.

Although we cannot determine causality for the serotonin-nigral iron association, several plausible scenarios merit consideration. The most enticing, given the association even in early stage disease, is that serotonergic dysfunction contributes to iron accumulation in the brain, particularly in the nigra. Murine models have been used to show that the serotonin transporter (SERT) regulates ventral midbrain iron concentrations^39,50^. There are several factors increased in PD that could cause SERT dysfunction (⍺-synuclein pathology^41^, TNF-⍺^42,51^^52^, SSRI/SNRI use^53^), and SERT dysfunction could, in turn, affect plasma serotonin levels; however, the effects of SERT dysfunction on plasma serotonin are nuanced^54,55^, and the mechanisms connecting SERT to iron remain unclear. An interesting possibility is that SERT is only one of multiple points of intersection between the serotonin system and iron regulation in the brain. Serotonin synthesis is iron-dependent^56^, and early iron deficiency in rodent models causes persistent changes in brain serotonin levels^57,58^. A serotonin-iron feedback loop, therefore, is plausible, and there is initial evidence that serotonin regulates iron directly, as activation of 5HT1C receptors increases transferrin production in choroid plexus epithelium^59^.

It also is possible that brain iron accumulation might lead directly to serotonergic neurodegeneration and dysfunction^37^) or that the relationship is indirect or mediated by a third or multiple factors. For instance, ⍺-synuclein and inflammation each have intricate, bidirectional ties to both serotonin and iron regulation and are suggested as pathogenic factors in PD^19,31,42,60–62^ Inflammation in particular stands out as a potential causal mechanism linking peripheral serotonin deficiency and brain iron accumulation. Serotonin has peripheral immunomodulatory actions contributing to both innate and adaptive immunity, and the immune system communicates with the brain via both humoral and neuronal mechanisms^63^. In addition, there is evidence that serotonin and other neuromodulators decrease microglial activation in the CNS^64^, the latter a process reported to be increased in both PD and Alzheimer’s disease and linked directly to regional iron dyshomeostasis and iron transport into the brain^17^. It would be useful to determine whether low serotonin and higher nigral iron cluster together with more extensive ⍺-synuclein pathology or signs of inflammation in a subset, or subtype, of patients.

This study had several strengths, including the large, clinically well-characterized PD Biomarker Program cohort^65^ and use of QSM, currently the most sensitive and reproducible MRI-based method for iron estimation^6,9,21–23,66,67^. One potential limitation was the use of plasma serotonin concentrations as a surrogate for brain or CSF levels. Serotonin is synthesized both centrally and peripherally, and plasma and CSF pools are separated by the blood–brain and blood-nerve barriers; thus, the relationship between plasma serotonin and nigral iron is likely to be indirect. Nevertheless, peripheral and central serotonin share common regulatory mechanisms (e.g., tryptophan hydroxylase, SERT), and plasma (or serum) and CSF serotonin concentrations are correlated^68^. Both low plasma and CSF serotonin concentrations have been associated with depression in PD in independent studies^26,37^, suggesting that a mechanistic link is possible. We suggest the association between low plasma serotonin and higher nigral iron in PD reflects a broader, more general relationship between systemic serotonin deficiency (or its upstream factors) and iron accumulation in the SNc and several other brain regions, but further studies are needed to test this hypothesis.

In our analysis, we understood that the source of a blood-derived sample (i.e., whole blood, platelets, platelet-rich or platelet-poor plasma) has a marked influence on serotonin concentrations, with the vast majority of serotonin in the blood being stored in platelets^68–70^. Although we used rigorous, standardized PDBP protocols for total plasma collection, we did not measure serotonin in these various compartments or completely deplete the plasma of platelets. Conversely, all samples were collected in identical fashion, and our whole-blood platelet counts did not differ between PD patients and controls (consistent with^71^) or influence the association between plasma serotonin and iron in the SNc. In the future, it might be useful to assess serotonin concentrations in the various blood compartments.

Future studies should also assess the high rate of SSRI/SNRI use in PD patients and its effect on plasma serotonin. About a third (35%) of PD patients have clinically significant depression^34^. In line with this, in the present cohort, approximately one-third of PD patients showed abnormally low plasma serotonin concentrations and/or were being treated with SSRI or SNRI antidepressants. There was significant overlap between these two groups. Due to the cross-sectional nature of this study, we could not determine whether low serotonin in any given individual existed prior to the use of antidepressant medications and might have contributed to depression and thus higher likelihood of antidepressant treatment or if low plasma serotonin was caused or exacerbated by SSRI/SNRI use. In prior studies, low serotonin (or 5-HIAA) in both plasma and CSF has been demonstrated in PD patients either not taking SSRI or SNRI medications^28,35,36^ or not taking any serotonergic agents^26,37,38^. However, two recent studies in the general population report that long-term SSRI treatment lowers plasma serotonin concentrations, and lower pretreatment plasma serotonin concentrations (and less change) predict poor treatment response^54,55^. Importantly, regardless of cause, the association of low plasma serotonin with higher nigral iron remained significant even after controlling for SSRI/SNRI use. Moreover, the correlation between plasma serotonin and nigral QSM values even within SSRI/SNRI users as a subgroup argues against the association being a simple group effect. Nevertheless, future studies should account for SSRI/SNRI use and investigate the underlying causes of low plasma serotonin in PD patients.

Aside from SSRI/SNRI use, other possible causes of low serotonin should be considered in future work. These include (1) increased inflammation-induced kynurenine pathway activation that leads to reduced amounts of tryptophan available for serotonin synthesis (recently reported in PD^72^), (2) changes in the microbiome that have been suggested to play a role in PD^73^ and might alter serotonin synthesis in enterochromaffin cells of the intestine^74^, and (3) altered catabolism of serotonin, a factor recently associated with hyperserotonemia in autism^75^.

This study also opens the door to mechanistic studies using animal or cellular models aimed at determining whether there is a causal relationship between low serotonin and iron accumulation in the brain in PD and, if so, any therapeutic opportunity. It would be interesting to explore the effects of various serotonergic agents, such as SSRIs, SNRIs, and long-acting 5HTP^76^, on regional iron content in the brain. Similarly, one could explore the effects of iron chelators on serotonin levels. Future human studies could also investigate the relationship between increased nigral iron and dopaminergic neuron loss in low serotonin patients (e.g., using PET, SPECT).

## Conclusions

This study revealed a robust correlation between low plasma serotonin and higher iron content in the SNc in PD, which was absent in controls. This correlation was present even in patients within one year of diagnosis, suggesting it might also be present in the prodromal phase, and was stronger in the SNc than any other brain region examined. Low serotonin and higher nigral iron were associated with a common set of clinical outcomes, and both were most strongly associated with SSRI/SNRI use, or treatment for depression/anxiety. Importantly, their correlation with each other persisted after controlling for this factor. We propose that these findings lend further support for a serotonin-iron relationship in the PD process and indicate a potential biochemical basis for the link between depression and nigral iron accumulation in PD patients recently reported^43^. Further studies in both patients and carefully selected and rigorously used animal models can determine direct mechanistic links if they exist as suggested by our data.

## Methods

### Participants

The study included 89 control and 97 PD subjects who participated in the National Institute of Neurological Disorders and Stroke PD Biomarker Program (NINDS PDBP) at the Pennsylvania State University between 2012 and 2015. PD patients were recruited from a tertiary movement disorders clinic, whereas controls were recruited from spouses and the local community. PD diagnosis was determined by a movement disorder specialist according to UK brain bank criteria. The enrollment criteria for PD subjects included history of adequate response to dopaminergic therapy, history of asymmetric onset, and lack of neurological disorders other than PD. PD duration was defined as the date since first PD diagnosis by a physician. Disease stages were set as early (< 1 year), mid (< 5 years), and late (5 + years) in accordance with previously established cutoffs^77^. All controls were free of known major medical and/or neurological conditions. All subjects provided written informed consent. The study was carried out in accordance with the Declaration of Helsinki. Ethical approval for the study was provided by the Pennsylvania State University Hershey Institutional Review Board.

### Blood sample collection and processing

Blood samples were obtained and processed in compliance with the PDBP laboratory manual. Blood samples were collected before 10:00 AM following an 8–12 h overnight fast using BD *Vacutainer* glass blood collection tubes with K_3_EDTA*.* Within 30 min of blood draw, samples were centrifuged at 1,500 × *g* for 15 min at 4 °C followed by aspiration and division of plasma into 1 mL aliquots prior to freezing at − 80° C. Care was taken not to disturb the buffy coat. Plasma samples were maintained at − 80° C for 4–7 years (with CO_2_ backup and 24/7/365 monitoring) prior to thawing once for sub-division of sample to send to the Van Andel Institute on dry ice for analysis. For analysis of peripheral serum iron metrics (red blood cell count, hemoglobin, hematocrit, serum iron, transferrin, total iron binding capacity (TIBC), transferrin saturation) and platelet counts, blood was collected into a BD *PST II* (plasma separator) tube and analyzed by the Penn State Hershey clinical lab using standard assays. This tube is routinely used in the clinical setting to measure iron, as these tubes lack EDTA, which is a known divalent metal chelator.

### Plasma serotonin measurement

Plasma serotonin was measured as described in^72^. Plasma serotonin concentrations were measured using a Waters Acquity ultra-high-performance liquid chromatography (UPLC) I class and Xevo triple-quadrupole mass spectrometry (TQ-S MS/MS) system. 10 µL samples were injected into an Acquity HSS T3 column conjugated with a Vanguard HSS T3 guard column and eluted using a mobile phase, 0.6% formic acid in Milli-Q water (Solvent A) and 0.6% formic acid in analytical grade methanol (Solvent B), at an isocratic flow rate of 0.3 mL/min. The intra-assay coefficient of variability for serotonin was 5.5%.

### Estimation of regional iron content using QSM and R2*

Brain MRI scans were offered to all subjects but completed by only a subset (70 controls, 62 PD patients). For each subject, T1-weighted, T2-weighted, and multi-gradient-echo MRIs were acquired using a 3 T Siemens scanner. Iron content was estimated using QSM (primary estimate of iron) and the transverse relaxation rate (R2*) since R2* has been suggested to capture brain tissue properties beyond iron^23^. QSM and R2* values were generated for the purposes of a prior study; detailed scan parameters, segmentation methods, and quantification methods can be found therein^78^. Mean QSM and R2* values were quantified for both sides of each of the following iron-rich or basal ganglia structures: SNc (for brevity referred to as the nigra), substantia nigra pars reticulata (SNr), putamen, globus pallidus, subthalamic nucleus, dentate nucleus, caudate nucleus, red nucleus, and thalamus. Regional QSM values were used to assess group differences in brain iron content and its relationship with plasma serotonin and other clinical variables.

### Clinical measures

NINDS common data elements were used to characterize participants clinically according to the PDBP protocol. PD-related metrics included the Movement Disorder Society-Unified Parkinson’s Disease Rating Scale (MDS-UPDRS), which consists of four subscales: non-motor aspects of daily living (UPDRS-I), motor aspects of daily living reported by the subjects (UPDRS-II), motor symptoms assessed by a trained examiner (UPDRS-III), and motor complications (UPDRS-IV). The UPDRS-III was administered in the “ON” state. Parkinson’s Disease Questionnaire 39 (PDQ-39), University of Pennsylvania Smell Identification Test (UPSIT), and Freezing of Gait Questionnaire (FOG-Q) scores also were obtained. Depression/behavioral metrics included the Hamilton Depression Rating Scale (HDRS), Hamilton Anxiety Rating Scale (HARS), Montreal Cognitive Assessment (MoCA), and Epworth Sleepiness Scale (ESS). Prescribed medications and indications were self-reported. Levodopa equivalent daily dose (LEDD) was calculated for PD subjects according to published criteria^79^. Selective serotonin (or serotonin and norepinephrine) reuptake inhibitor (SSRI/SNRI) antidepressant use (yes/no) served as an indication for treatment of depression/anxiety.

### Statistical methods

Statistical analyses were performed using IBM’s SPSS (version 26) and SAS (version 9.4). All figures were produced using the ggplot2 package^80^ in R (version 3.5.3)^81^. Plasma serotonin and nigral QSM and R2* distributions first were tested for normality, with plasma serotonin distributions failing this test (Shapiro–Wilk, *p* < 0.001). Plasma serotonin concentrations thus underwent logarithmic transformation (natural log) prior to parametric analyses. Control and Parkinson’s subjects were compared using independent *t*-tests, ANCOVA with age and sex as covariates, or chi-square tests, as appropriate. Two-tailed tests were used to determine the statistical significance at ⍺ = 0.05. All mean values are reported ± standard deviation. Because the measurement of iron in brain regions other than the SNc and clinical metrics was exploratory, multiple comparison corrections were not performed.

Associations between plasma serotonin and QSM, R2*, and serum iron markers were determined using Pearson’s correlation coefficients and partial Pearson’s correlations, controlling for potential confounders. Associations between plasma serotonin and clinical measures within PD patients were first assessed using Pearson’s correlations, then in more depth using linear regression models (Type III). Two models were used to compare these associations. Model 1a and 1b tested the association between plasma serotonin and each clinical measure in all PD patients (*n* = 97) and in the subset with MRI data (*n* = 62). Model 2 tested this association in the subset with MRI data also controlling for SNc QSM (*n* = 62). All models controlled for age, sex, and disease stage as factors, and were run with only main effects and also with main effects plus the plasma serotonin and sex interaction. Since no interaction was observed, we only presented main effects models.

## Supplementary Information

Supplementary Information.
