# Supplementary material for: Low plasma serotonin linked to higher nigral iron in Parkinson’s disease
Source: Sci Rep. 2021 Dec 21;11:24384. doi: 10.1038/s41598-021-03700-2 (PMC8692322; doi:10.1038/s41598-021-03700-2)
Supplement: Supplementary file 1 — Supplementary Information. [file 41598_2021_3700_MOESM1_ESM.docx]

# Supplementary Information

## Supplementary Fig. S1.


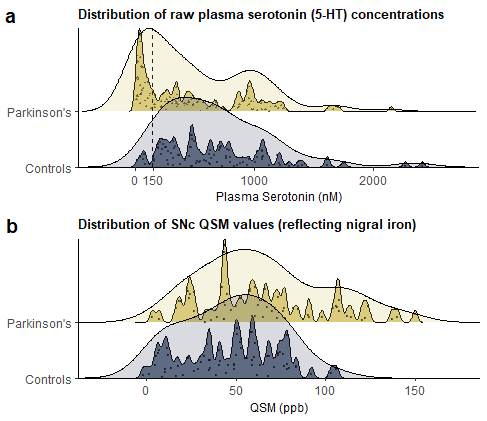


**Supplemental Fig. S1. Distributions of raw plasma serotonin concentrations and QSM values in the substantia nigra pars compacta in Parkinson’s subjects (PD) and controls.** Large and small bandwidth density plots are overlaid. Data points represent individual subjects. In (a), the vertical dashed line marking the sharp peak in the Parkinson’s plasma serotonin distribution indicates the lowest tertile of Parkinson’s subjects, which corresponds to concentrations <150 nM. Due to the prominent cluster of Parkinson’s patients with concentrations below this cutoff, this tertile is marked in all other plasma serotonin plots for reference. Figure was produced using the ggplot2 package^80^ in R (version 3.5.3)^81^.

## Supplementary Fig. S2.

**
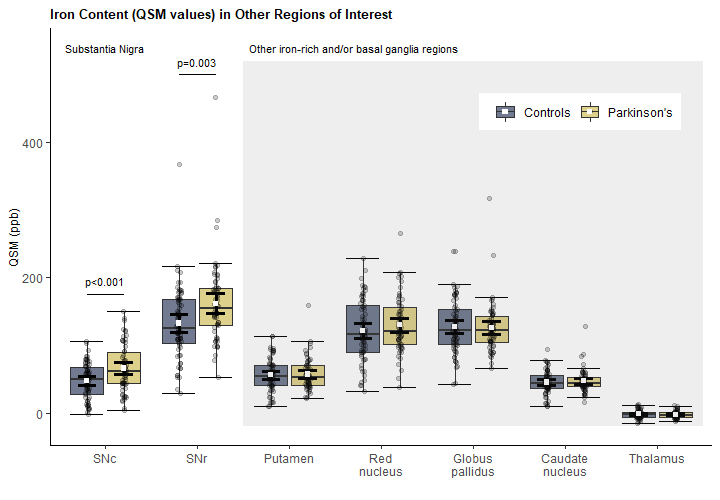
**

**Supplementary Fig. S2. QSM values, reflecting iron content, in Parkinson’s patients vs. controls by region.** P-values indicate results of ANCOVA (performed per region) with age and sex as covariates (p-values >0.05 not shown). White box and error bars indicate age and sex-adjusted mean and 95% CI. Figure was produced using the ggplot2 package^80^ in R (version 3.5.3)^81^.

## Supplementary Table S1.

|  | Group Difference | | | Correlation with  Plasma Serotonin in Parkinson’s | |
| --- | --- | --- | --- | --- | --- |
|  | Controls | Parkinson’s | P-value | Partial R | P-value |
| Red blood cells | 4.6 ± 0.40 | 4.6 ± 0.45 | 0.998 | 0.025 | 0.810 |
| Hemoglobin | 13.8 ± 1.2 | 13.8 ± 1.3 | 0.931 | 0.079 | 0.451 |
| Hematocrit | 41.0 ± 3.2 | 41.0 ± 3.3 | 0.956 | 0.080 | 0.445 |
| Iron | 94.5 ± 32.6 | 100.9 ± 33.4 | 0.190 | 0.056 | 0.592 |
| Transferrin | 257.3 ± 40.2 | 253.5 ± 33.6 | 0.483 | -0.142 | 0.176 |
| TIBC | 322.9 ± 47.9 | 317.5 ± 37.7 | 0.395 | -0.164 | 0.117 |
| Iron Saturation | 30.5 ± 14.3 | 32.2 ± 12.2 | 0.390 | 0.097 | 0.354 |

**Supplementary Table S1.** **Serum iron values in Parkinson’s and control subjects and their correlation with plasma serotonin in Parkinson’s disease.** Data reflect group means ± standard deviation for serum iron markers in Parkinson’s disease and control subjects. P values represent the results of independent samples t-tests (two-tailed significance). Partial correlation analyses tested the association of each marker with plasma serotonin in Parkinson’s patients, after controlling for age and sex. For all correlations, df=91. P-values are not corrected for multiple comparisons.

## Supplementary Table S2.

| Region | Controls (n=70) | | Parkinson’s (n=62) | |
| --- | --- | --- | --- | --- |
|  | Pearson’s R | P-value | Pearson’s R | P-value |
| Putamen | -0.134 | 0.270 | **-0.396** | **0.001** |
| Substantia nigra pars reticulata | -0.099 | 0.414 | **-0.315** | **0.013** |
| Globus pallidus | -0.041 | 0.739 | **-0.292** | **0.021** |
| Subthalamic nucleus | -0.097 | 0.424 | **-0.289** | **0.023** |
| Dentate nucleus | 0.003 | 0.982 | **-0.254** | **0.046** |
| Red nucleus | -0.027 | 0.823 | -0.234 | 0.067 |
| Caudate nucleus | -0.131 | 0.279 | -0.177 | 0.168 |
| Thalamus | -0.201 | 0.094 | -0.051 | 0.694 |

**Supplementary Table S2. Correlations between plasma serotonin and QSM values in regions other than the SNc.** Pearson’s correlation coefficients and p-values are presented reflecting the correlation between plasma serotonin and QSM in each brain region in control subjects and Parkinson’s patients. P-values are not corrected for multiple comparisons.

## Supplementary Table S3.

|  | | Estimate | SE | t ^a^ | **P-value** |
| --- | --- | --- | --- | --- | --- |
| **UPDRS I** | | | | | |
| Model 1a (entire cohort; n=97) | ln(5-HT) | -1.03 | 0.52 | -1.99 | **0.049** |
| Model 1b (MRI cohort; n=62) | ln(5-HT) | -1.10 | 0.66 | -1.65 | 0.104 |
| Model 2 (MRI cohort, n=62,  both 5-HT and QSM as predictors) | ln(5-HT) | -0.61 | 0.74 | -0.83 | 0.411 |
|  | QSM in SNc | 0.04 | 0.03 | 1.49 | 0.142 |
| **Parkinson’s Disease Questionnaire (PDQ)** | | | | | |
| Model 1a (n=97) | ln(5-HT) | -4.51 | 1.70 | -2.65 | **0.010** |
| Model 1b (n=62) | ln(5-HT) | -3.77 | 2.20 | -1.71 | 0.092 |
| Model 2 (n=62) | ln(5-HT) | -2.69 | 2.46 | -1.09 | 0.279 |
|  | QSM in SNc | 0.09 | 0.09 | 0.98 | 0.330 |
| **Freezing of Gait Questionnaire (FOG-Q)** | | | | | |
| Model 1a (n=97) | ln(5-HT) | -0.83 | 0.37 | -2.24 | **0.028** |
| Model 1b (n=62) | ln(5-HT) | -0.88 | 0.49 | -1.78 | 0.080 |
| Model 2 (n=62) | ln(5-HT) | -0.61 | 0.55 | -1.11 | 0.270 |
|  | QSM in SNc | 0.02 | 0.02 | 1.08 | 0.283 |
| **LEDD** | | | | | |
| Model 1a (n=97) | ln(5-HT) | -68.6 | 26.2 | -2.62 | **0.010** |
| Model 1b (n=62) | ln(5-HT) | -107.1 | 34.0 | -3.15 | **0.003** |
| Model 2 (n=62) | ln(5-HT) | -84.0 | 37.7 | -2.23 | **0.030** |
|  | QSM in SNc | 1.82 | 1.33 | 1.37 | 0.177 |

# Supplementary Table S3. For each clinical metric, the results of two regression models are shown depicting Model 1a) the association between each clinical metric and plasma serotonin using full Parkinson’s cohort (n=97) Model 1b) the same association but using the MRI subset (n=62) Model 2) the association in Model 1b also accounting for QSM in the substantia nigra pars compacta (SNc). Data presented are the model estimate, standard error, t from the regression, and p value. All models included the covariates age, sex, and disease stage.
